# Maternal cardiometabolic markers are associated with fetal growth: a secondary exploratory analysis of the LIMIT randomised trial

**DOI:** 10.1186/s12902-019-0416-x

**Published:** 2019-10-10

**Authors:** Cecelia M. O’Brien, Jennie Louise, Andrea Deussen, Jodie M. Dodd

**Affiliations:** 1 School of Paediatrics and Reproductive Health, and Robinson Research Institute, University of Adelaide, Adelaide, Australia; 2 School of Public Health, University of Adelaide, Adelaide, Australia; 3 Department of Perinatal Medicine, Women’s and Babies Division, Women’s and Children’s Hospital, Adelaide, Australia; 4 Women’s and Children’s Hospital, The University of Adelaide, 72 King William Road, North Adelaide, SA 5006 Australia

**Keywords:** Obesity, Pregnancy, Cardiometabolic markers, Adiponectin, Fetal body composition

## Abstract

**Background:**

To determine the association between maternal cardiometabolic and inflammatory markers with measures of fetal biometry and adiposity.

**Methods:**

Women included in this exploratory analysis were randomised to the ‘Standard Care’ group (*N* = 911) from the LIMIT randomised trial involving a total of 2212 pregnant women who were overweight or obese (ACTRN12607000161426, Date of registration 9/03/2007, prospectively registered). Fetal biometry including abdominal circumference (AC), estimated fetal weight (EFW), and adiposity measurements (mid-thigh fat mass, subscapular fat mass, abdominal fat mass) were obtained from ultrasound assessments at 28 and 36 weeks’ gestation. Maternal markers included C reactive protein (CRP), leptin and adiponectin concentrations, measured at 28 and 36 weeks’ gestation and fasting triglycerides and glucose concentrations measured at 28 weeks’ gestation.

**Results:**

There were negative associations identified between maternal serum adiponectin and fetal ultrasound markers of biometry and adiposity. After adjusting for confounders, a 1-unit increase in log Adiponectin was associated with a reduction in the mean AC z score [− 0.21 (− 0.35, − 0.07), *P* = 0.004] and EFW [− 0.23 (− 0.37, − 0.10), *P* < 0.001] at 28 weeks gestation. Similarly, a 1-unit increase in log Adiponectin was association with a reduction in the mean AC z score [− 0.30 (− 0.46, − 0.13), *P* < 0.001] and EFW [− 0.24 (− 0.38, − 0.10), *P* < 0.001] at 36 weeks gestation. There were no consistent associations between maternal cardiometabolic and inflammatory markers with measurements of fetal adiposity.

**Conclusion:**

Adiponectin concentrations are associated with measures of fetal growth. Our findings contribute to further understanding of fetal growth in the setting of women who are overweight or obesity.

## Background

Obesity represents a major global health challenge, with over 50% of women entering pregnancy in high-income countries with a body mass index (BMI) greater than 25 kg/m^2^ [1, 2]. There are well-recognised associations between obesity in pregnancy and maternal, fetal and neonatal health outcomes [3]. In the long-term, there are clear associations between maternal obesity, fetal overgrowth, high infant birth weight, and subsequent childhood obesity [4].

In 1969, Pedersen postulated that maternal hyperglycaemia stimulates hyperinsulinemia in the fetus, which in turn directly stimulates fetal growth through insulin growth factors [5]. In the past 10 years, there has been increasing recognition of the ‘indirect’ pathway, involving leptin, adiponectin, triglycerides, cholesterol and inflammatory cytokines which is mediated via placental transfer [6]. Furthermore, maternal obesity during critical time points for fetal development has been linked to fetal programming via epigenetic modification [7].

During pregnancy, triglycerides and fatty acids are required for fetal development and growth [8]. Lipoprotein receptors, binding proteins and lipases hydrolyse triglycerides to free fatty acids for uptake by the syncytiotrophoblast, enabling transportation to the fetus along with storage and metabolism within the placenta [9, 10]. Enhanced placental lipid transport in obese women has been hypothesised [11] but has not been demonstrated [12, 13]. Higher concentrations of triglycerides and lipids have been found in women who are obese [12] and those women who have delivered a large for gestational age infant [9, 14]. Studies investigating newborn cord blood concentrations of lipoproteins [15] have shown an association with adipose tissue in the fetus and newborn, contributing to higher infant birth weight [9] and neonatal adiposity [16].

A key component of the fetal overgrowth pathway is adiponectin, the endocrine link between maternal adipose tissue and fetal growth [17]. Adiponectin is secreted by maternal adipocytes, acting directly on the placenta without crossing into the fetal circulation [17, 18]. While adiponectin is known to be insulin sensitising in the skeletal muscle and liver [19], it exerts the opposite effect in the placenta [20]. During pregnancy, adiponectin inhibits insulin mediated amino acid transport in trophoblast cells via the insulin receptor (IRS) and mTORC1 signalling [19, 20]. As gestation advances, adiponectin levels decline due to the physiological resistance to insulin in maintaining serum glucose [21]. In non-pregnant adults, adiponectin concentrations are lower in obesity [22], cardiovascular disease [18] and Type 2 Diabetes mellitus [23]. During pregnancy, lower concentration of adiponectin is associated with gestational diabetes [24]. Maternal and fetal adiponectin appear to have opposite effects in relation to fetal growth [17], with low maternal concentrations of adiponectin stimulating fetal overgrowth [22]. Conversely, cord blood and neonatal adiponectin concentrations have been reported to be up to 7 times higher than maternal concentrations, with positive correlations with infant birth weight [25] and anthropometric measures of neonatal adiposity [25, 26].

With the widespread availability and technological advances in fetal ultrasound, there is growing interest in the measurement and prediction of fetal overgrowth and adiposity [27]. However, the current literature is limited to relatively small sample sizes and mostly involving women entering pregnancy with a normal BMI [28–31].

The aim of this secondary exploratory analysis was to determine if maternal cardiometabolic and inflammatory markers were associated with fetal growth and adiposity measured by ultrasound in women who are overweight or obese in pregnancy at 28 and 36 weeks gestation.

## Methods

The research methodology [32] and clinical findings [33] of the LIMIT randomised controlled trial have been published previously. The conduct of the LIMIT randomised trial adhered to CONSORT methodology [34]. Women with a BMI ≥25 kg/m^2^, singleton pregnancy, without a diagnosis of diabetes and between 10 + 0 and 20 + 0 weeks gestation were recruited between June 2008 and December 2011 from 3 public hospitals across metropolitan Adelaide. The women were randomised to either the ‘Lifestyle Advice Group’, receiving standard antenatal care and diet and lifestyle, or ‘Standard Care Group’ receiving standard antenatal care without additional diet and lifestyle advice. The intervention was delivered by a research dietician and trained research assistants. Further details regarding content of the intervention [33, 35] and the fetal growth study [36, 37] have been previously published. Women included in this analysis were those randomised to the Standard Care Group.

### Measurement of cardiometabolic and inflammatory markers

Maternal blood samples were obtained at 28 and 36 weeks gestation, and cord blood was obtained at birth and the methodology has been previously described in detail [38]. At 28 weeks, a fasting maternal serum sample was collected for all participants in the LIMIT trial. The following cardiometabolic markers were measured; total cholesterol, triglycerides, non-esterified fatty acids (NEFA), high-density lipoprotein cholesterol, insulin, glucose, leptin, adiponectin and C reactive protein. The majority (glucose, cholesterol, HDL-C, triglycerides, NEFA and CRP) were measured using Roche Diagnostics commercial kits (Australia) and non-esterified fatty acids were measured using Wako Pure Chemical Industries (Japan). All assays were performed on the automated Hitachi Auto 912 analyser or Cobas Integra 400 Plus with appropriate calibrators and quality controls (Roche for Roche assays and Wako standard and Sero QC’s for the NEFA C assay). Plasma leptin (in singulate; HL-81 K; Millipore, St. Charles, MO, USA) and adiponectin (in singulate; HADP-61HK; Millipore, St. Charles, MO, USA) were determined by double antibody radioimmunoassay following the methods from the supplier.

At 36 weeks, a non-fasting maternal serum sample was collected and total cholesterol, triglycerides, non-esterified fatty acids (NEFA), high-density lipoprotein cholesterol, insulin, glucose, leptin, adiponectin and C reactive protein were measured.

### Ultrasound assessment

Women were offered a research ultrasound scan at approximately 28 and 36 weeks’ gestation, at which time fetal biometry, wellbeing and body composition measurements were obtained as previously described [36, 37]. Estimated date of confinement was verified for all women based on last menstrual period and early ultrasound scan. All measurements were obtained prospectively by medical practitioners with specialist or subspecialist training in obstetric ultrasound whilst blinded to the woman’s research treatment allocation.

#### Ultrasound outcome measurements

##### Biometry and estimated fetal weight

Fetal biometry included head circumference, biparietal diameter, abdominal circumference and femur length, measured in accordance with national and international standards of practice [39]. Estimated fetal weight was calculated using the Hadlock C formula [40].

##### Fetal body composition measurements

Mid-thigh fat mass (MTFM) and lean mass (MTLM), abdominal fat mass (AFM) and sub-scapular fat mass (SSFM) were measured using techniques that have been published previously [36, 37]. Grivell and associates also reported the inter-observer variability for adiposity measures and found moderate agreement demonstrated for SSFM, MTTM, MTFM and fair agreement for AFM and MTLM [37].

##### Mid-thigh total, lean and fat mass

MTLM was calculated by obtaining a longitudinal view of the femur and identification of the midpoint at a zero degree angle. The transducer was rotated through 90 degrees to obtain a cross sectional view of the mid-thigh. A trace of the circumference of the MTTM was performed and area was calculated, followed by the MTLM incorporating muscle and bone. A subtraction was performed between the MTTM and the MTLM to calculate the mid-thigh fat mass (MTFM).

##### Abdominal fat mass

Abdominal fat mass or anterior abdominal wall thickness was obtained between the mid-axillary lines and anterior to the margins of the ribs, at the level of the abdominal circumference. The subcutaneous fat is represented by the echogenic envelope surrounding the abdomen and is measured in millimetres. Using magnification, 4 measurements were obtained from one or two separate images, and the mean was used in the analysis.

##### Subscapular fat mass

Using a sagittal view of the fetal trunk, the entire longitudinal section of the scapular was located between the skin surface and the subcutaneous tissue at the interface with the super-spinous and infra-spinous muscles. Two measurements of the subcutaneous skin width at the end of the bone were taken and the mean value was used in the analysis.

#### Statistical analysis

Baseline characteristics of women in the Standard Care group were assessed descriptively. Normally distributed continuous variables are reported as mean and standard deviation or median and interquartile range as appropriate. Categorical variables are reported as a number and percentage and the chi squared statistic was used accordingly.

For each fetal biometry measured, z scores were calculated using ultrasound growth charts in clinical use [40]. All cardiometabolic markers were log transformed prior to analysis due to skewed distributions. Estimates are back-transformed to the original scale and therefore represent ratios of geometric means (approximately ratios of medians).

The investigation concerns cross-sectional relationships, i.e. whether there is an association between cardiometabolic/inflammatory markers at 28 weeks, and fetal ultrasound measures at 28 weeks (and similarly for 36 weeks). Because the nature of the association was of interest, and because most of the cardiometabolic/inflammatory markers exhibited skewness in distribution, each of the cardiometabolic/inflammatory markers was log-transformed prior to analysis. Estimates represent the difference in mean fetal measure corresponding to a 1-unit increase in log cardiometabolic marker. For example at 28 weeks’ gestation, a 1 unit increase in log CRP corresponds to a decrease in mean EFW of 8.62 (29.88, 12.63) grams (*p* = 0.426).

Three of the cardiometabolic/inflammatory markers (CRP, leptin and adiponectin) were measured at both 28 and 36 weeks. For these markers, linear regression models were used to model the relationship between the marker and fetal ultrasound measures at each time point, including a time-by- marker interaction term to test whether the relationship differs between time points. Generalised Estimating Equations (GEEs) with exchangeable working correlation were used to account for repeated measures. Triglycerides and fasting glucose were measured at 28 weeks only; therefore, for these markers, relationships with 28 week fetal ultrasound measures only were investigated using linear regression models.

Both unadjusted and adjusted analyses were performed, with the adjusted analyses including study centre, parity (0 vs ≥1), maternal BMI category (25.0–29.9 vs ≥30.0), smoking status, SEIFA IRSD quintile, and age at consent as covariates.

Although both fetal biometry and adiposity measures and maternal cardiometabolic and inflammatory markers varied over time, standard linear regression models with GEEs were considered appropriate to model the associations, as no causal interpretation of the associations was intended, and there is additionally no plausible pathway by which the fetal biometry and adiposity outcomes at the earlier time point could influence the value of maternal cardiometabolic markers at a later time point.

Statistical significance was assessed at the two sided *P* < 0.05 and no adjustment was made for multiple comparisons. All analyses were performed using SAS 9.4 (Cary, NC, USA).

## Results

### Demographic characteristics

**Fig. 1:**
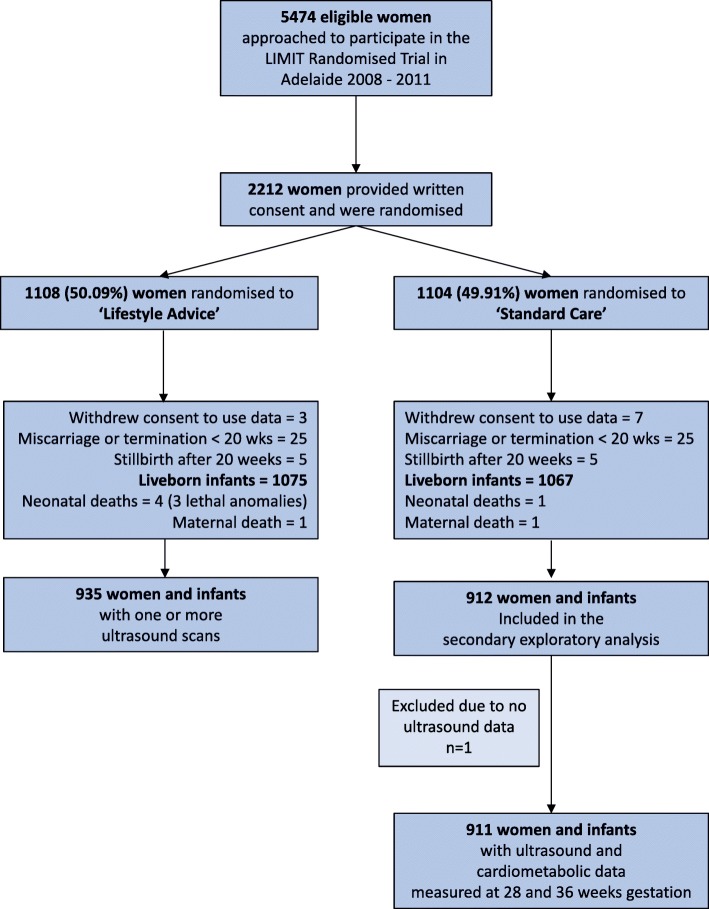
CONSORT diagram detailing the participant flow in the LIMIT trial and this secondary analysis

This secondary exploratory analysis included a total of 1104 women, who were randomised to the ‘Standard Care’ group of the LIMIT randomised controlled trial. Of these women, 912 women had a minimum of one ultrasound performed at 28 or 36 weeks and one woman was excluded from this analysis due to incomplete ultrasound data (Fig. 1). Table 1 summarises the baseline characteristics of the 911 women who participated in these analyses. Mean maternal age was 29.6 years (standard deviation 5.5) with 41% of women (*n* = 377) overweight, 46.5% (*n* = 424) obese (BMI 30–39.9 kg/m^2^), and 12.2% (*n* = 111) morbidly obese, with BMI greater than 40 kg/m^2^. Most women (92%, *n* = 835) were of Caucasian origin, 40% (*n* = 369) were in their first ongoing pregnancy, and approximately 30% (*n* = 265) were from the highest quintile of social disadvantage. The baseline characteristics of the women contributing ultrasound data were comparable to all women in the standard care group, and to the full randomized LIMIT cohort [33].

**Table 1:** Baseline characteristics of the Standard Care group within the LIMIT Trial

	Description
Number (*N*)	911
Body Mass Index Mean (SD)	32.60 (6.01)
BMI Category	
25–29.9 kg/m2	377 (41.3)
30–34.9 kg/m2	271 (29.7)
35–39.9 kg/m2	153 (16.8)
> 40 kg/m2	111 (12.2)
Maternal Age (years) Mean (SD)	29.6 (5.5)
Nulliparous *n* (%)	369 (40.5)
Smoker *n* (%)	101 (11.1)
SEIFA Quintiles Mean (SD)	
*Quintile 1* Most disadvantaged	265 (29.06)
*Quintile 2*	223 (24.45)
*Quintile 3*	143 (15.68)
*Quintile 4*	142 (15.57)
*Quintile 5* Least disadvantaged	139 (15.24)
Caucasian *n* (%)	836 (91.67)

### C-reactive protein (CRP)

No consistent associations were found between serum CRP concentrations and fetal ultrasound measures of biometry and adiposity (Table 2).

**Table 2:** Relationship between log CRP and Fetal ultrasound markers

Ultrasound Measure	Unadjusted Estimate (95% CI)	Unadjusted *p* value	Adjusted Estimate (95% CI)	Adjusted *p* value
EFW		0.559*		0.835*
- 28 Weeks	−6.41 (−26.19, 13.36)	0.525	−8.62 (−29.88, 12.63)	0.426
- 36 Weeks	−17.06 (−53.13, 19.01)	0.354	−12.50 (−47.91, 22.90)	0.489
SSFM		0.842*		0.850*
- 28 Weeks	−0.00 (− 0.10, 0.09)	0.920	0.02 (− 0.08, 0.11)	0.744
- 36 Weeks	0.01 (− 0.14, 0.16)	0.875	0.03 (−0.12, 0.18)	0.675
AFM		0.442*		0.394*
- 28 Weeks	0.00 (−0.10, 0.10)	0.990	−0.00 (− 0.11, 0.11)	0.976
- 36 Weeks	0.07 (−0.09, 0.23)	0.396	0.08 (−0.08, 0.23)	0.353
MTFM		0.988*		0.998*
- 28 Weeks	0.04 (−0.08, 0.17)	0.514	0.05 (−0.09, 0.19)	0.456
- 36 Weeks	0.04 (−0.29, 0.37)	0.817	0.05 (−0.28, 0.39)	0.758
MTLM		0.419*		0.376*
- 28 Weeks	0.04 (−0.06, 0.15)	0.414	0.05 (−0.06, 0.16)	0.367
- 36 Weeks	−0.05 (− 0.28, 0.17)	0.636	− 0.06 (− 0.28, 0.17)	0.615
AC		0.698*		0.998*
- 28 Weeks	−0.05 (− 0.20, 0.09)	0.473	− 0.07 (− 0.22, 0.08)	0.351
- 36 Weeks	− 0.09 (− 0.26, 0.08)	0.296	−0.07 (− 0.24, 0.10)	0.407
BPD		0.114*		0.147*
- 28 Weeks	− 0.03 (− 0.06, 0.00)	0.096	− 0.02 (− 0.06, 0.01)	0.167
- 36 Weeks	0.00 (− 0.03, 0.03)	0.883	0.00 (− 0.03, 0.04)	0.770
HC		0.682*		0.568*
- 28 Weeks	−0.07 (− 0.19, 0.05)	0.259	− 0.07 (− 0.19, 0.05)	0.280
- 36 Weeks	− 0.04 (− 0.15, 0.07)	0.462	−0.03 (− 0.13, 0.08)	0.625
FL		0.961*		0.824*
- 28 Weeks	− 0.01 (− 0.04, 0.02)	0.491	− 0.01 (− 0.04, 0.02)	0.428
- 36 Weeks	−0.01 (− 0.04, 0.02)	0.478	− 0.01 (− 0.04, 0.02)	0.602
EFW z score		0.466*		0.587*
- 28 Weeks	0.03 (− 0.04, 0.10)	0.380	0.02 (− 0.04, 0.09)	0.482
- 36 Weeks	− 0.00 (− 0.08, 0.08)	0.981	0.00 (− 0.08, 0.08)	0.979
AC z score		0.611*		0.791*
- 28 Weeks	0.02 (−0.06, 0.10)	0.609	0.01 (−0.07, 0.09)	0.808
- 36 Weeks	−0.01 (− 0.10, 0.09)	0.900	− 0.00 (− 0.09, 0.09)	0.931
BPD z score		0.095*		0.169*
- 28 Weeks	−0.05 (− 0.16, 0.06)	0.399	− 0.04 (− 0.15, 0.08)	0.544
- 36 Weeks	0.05 (− 0.04, 0.15)	0.261	0.05 (− 0.05, 0.15)	0.302
HC z score		0.706*		0.637*
- 28 Weeks	−0.00 (− 0.08, 0.08)	0.932	− 0.00 (− 0.09, 0.08)	0.914
- 36 Weeks	0.01 (− 0.06, 0.09)	0.738	0.02 (− 0.06, 0.09)	0.674
FL z score		0.942*		0.827*
- 28 Weeks	0.01 (−0.07, 0.09)	0.768	0.00 (−0.08, 0.09)	0.920
- 36 Weeks	0.02 (−0.07, 0.11)	0.726	0.02 (−0.08, 0.11)	0.738

Results are expressed as the expected difference (ratio) and 95% confidence intervals

Adjusted analyses including BMI category (< 30 vs ≥30), study centre, parity (0 vs ≥1), age at consent, smoking status and SEIFA IRSD quintile

* *p* value for test of time-by-log CRP interaction to test whether the association between fetal ultrasound measure and log CRP are different at 36 weeks to the association at 28 weeks

### Triglycerides

There were no consistent associations identified between serum triglyceride concentrations at 28 weeks and fetal ultrasound markers of biometry and adiposity (Table 3). However, there was a positive association identified between maternal serum triglyceride concentrations and biometry z-scores. Specifically, a 1-unit increase in log triglyceride concentration was associated with an increase in mean EFW z-score of 0.20 (0.01 to 0.39; *p* = 0.041), and an increase in mean AC z-score of 0.25 (0.05 to 0.46; *p* = 0.016).

**Table 3:** Relationship between log Triglycerides and Fetal ultrasound markers

Ultrasound Measure	Unadjusted Estimate (95% CI)	Unadjusted *p* value	Adjusted Estimate (95% CI)	Adjusted *p* value
EFW	26.96 (−19.21, 73.13)	0.252	28.14 (−18.38, 74.67)	0.236
SSFM	0.02 (− 0.21, 0.25)	0.870	0.06 (− 0.18, 0.29)	0.633
AFM	0.02 (− 0.23, 0.27)	0.899	0.01 (− 0.24, 0.26)	0.940
MTFM	− 0.01 (− 0.32, 0.31)	0.964	− 0.00 (− 0.32, 0.32)	> 0.99
MTLM	0.08 (− 0.19, 0.34)	0.567	0.04 (− 0.23, 0.31)	0.779
AC	0.27 (− 0.07, 0.62)	0.121	0.25 (− 0.10, 0.60)	0.154
BPD	0.03 (−0.06, 0.11)	0.540	0.04 (−0.04, 0.13)	0.334
HC	−0.01 (− 0.29, 0.27)	0.946	0.03 (− 0.26, 0.32)	0.851
FL	−0.01 (− 0.07, 0.06)	0.842	0.00 (− 0.06, 0.07)	0.908
EFW z score	0.23 (0.04, 0.42)	0.020	0.20 (0.01, 0.39)	0.041
AC z score	0.30 (0.09, 0.50)	0.004	0.25 (0.05, 0.46)	0.016
BPD z score	0.25 (− 0.06, 0.55)	0.113	0.29 (− 0.02, 0.59)	0.067
HC z score	0.03 (−0.18, 0.23)	0.808	0.03 (−0.18, 0.24)	0.796
FL z score	0.05 (− 0.15, 0.26)	0.612	0.07 (− 0.14, 0.28)	0.499

Results are expressed as the expected difference (ratio) and 95% confidence intervals

Adjusted analyses including BMI category (< 30 vs ≥30), study centre, parity (0 vs ≥1), age at consent, smoking status and SEIFA IRSD quintile

### Fasting glucose

There were no consistent associations found between fasting glucose concentrations at 28 weeks and fetal ultrasound measures of biometry and adiposity (Table 4).

**Table 4:** Relationship between log Fasting Glucose and Fetal ultrasound markers

Ultrasound Measure	Unadjusted Estimate (95% CI)	Unadjusted *p* value	Adjusted Estimate (95% CI)	Adjusted *p* value
EFW	59.68 (−33.80, 153.16)	0.211	53.52 (−56.64, 163.68)	0.341
SSFM	−0.32 (− 0.81, 0.17)	0.201	− 0.17 (− 0.74, 0.41)	0.575
AFM	− 0.54 (−1.10, 0.02)	0.058	− 0.10 (− 0.76, 0.57)	0.772
MTFM	− 0.25 (− 0.97, 0.47)	0.493	0.02 (− 0.83, 0.87)	0.959
MTLM	− 0.28 (− 0.91, 0.35)	0.387	0.03 (− 0.72, 0.77)	0.946
AC	0.16 (− 0.55, 0.88)	0.653	0.33 (− 0.50, 1.17)	0.430
BPD	0.16 (− 0.01, 0.33)	0.067	0.08 (− 0.13, 0.28)	0.460
HC	0.19 (−0.38, 0.77)	0.509	0.05 (−0.63, 0.74)	0.884
FL	0.12 (−0.01, 0.26)	0.077	0.09 (−0.07, 0.25)	0.289
EFW z score	0.33 (−0.09, 0.74)	0.120	0.46 (−0.01, 0.94)	0.057
AC z score	0.08 (−0.35, 0.51)	0.714	0.36 (−0.14, 0.87)	0.153
BPD z score	0.61 (−0.03, 1.25)	0.061	0.42 (−0.32, 1.17)	0.263
HC z score	0.10 (−0.34, 0.53)	0.659	0.07 (−0.45, 0.59)	0.800
FL z score	0.50 (0.06, 0.94)	0.027	0.49 (−0.04, 1.03)	0.071

Results are expressed as the expected difference (ratio) and 95% confidence intervals

Adjusted analyses including BMI category (< 30 vs ≥30), study centre, parity (0 vs ≥1), age at consent, smoking status and SEIFA IRSD quintile

### Leptin

There were no consistent associations identified between serum leptin concentrations and fetal ultrasound markers of biometry and adiposity (Table 5). However, there was a positive association identified between serum leptin concentration and mid-thigh fat mass (MTFM). Specifically, a 1-unit increase in *log* leptin concentration was associated with a greater reduction in mean MTFM of − 0.37 (− 0.67, − 0.07) at 28 weeks (*p* = 0.015).

**Table 5:** Relationship between log Leptin and Fetal ultrasound markers

Ultrasound Measure	Unadjusted Estimate (95% CI)	Unadjust *p* value	Adjusted Estimate (95% CI)	Adjusted *p* value
EFW		0.815		0.785*
- 28 Weeks	−40.68 (−79.26, −2.09)	0.039	−41.08 (− 83.65, 1.49)	0.059
- 36 Weeks	−32.84 (−95.88, 30.20)	0.307	−31.83 (−95.48, 31.83)	0.327
SSFM		0.925		0.999*
- 28 Weeks	0.01 (− 0.17, 0.20)	0.880	0.14 (− 0.06, 0.34)	0.167
- 36 Weeks	0.03 (− 0.24, 0.30)	0.833	0.14 (− 0.13, 0.41)	0.303
AFM		0.988		0.912*
- 28 Weeks	− 0.02 (− 0.22, 0.17)	0.814	0.03 (− 0.19, 0.24)	0.802
- 36 Weeks	− 0.02 (− 0.33, 0.28)	0.894	0.01 (− 0.30, 0.32)	0.960
MTFM		0.561		0.563
- 28 Weeks	− 0.25 (− 0.51, 0.00)	0.053	− 0.37 (− 0.67, − 0.07)	0.015*
- 36 Weeks	−0.08 (− 0.65, 0.49)	0.778	− 0.20 (− 0.76, 0.36)	0.488
MTLM		0.231		0.191*
- 28 Weeks	0.07 (−0.13, 0.27)	0.496	0.00 (−0.24, 0.24)	0.995
- 36 Weeks	−0.16 (− 0.50, 0.19)	0.373	− 0.25 (− 0.60, 0.10)	0.167
AC		0.705		0.631*
- 28 Weeks	−0.25 (− 0.52, 0.02)	0.065	− 0.26 (− 0.54, 0.02)	0.067
- 36 Weeks	− 0.19 (− 0.48, 0.11)	0.221	− 0.18 (− 0.48, 0.13)	0.259
BPD		0.400		0.420*
- 28 Weeks	− 0.02 (− 0.09, 0.05)	0.605	− 0.01 (− 0.08, 0.06)	0.735
- 36 Weeks	0.02 (− 0.04, 0.08)	0.614	0.02 (− 0.04, 0.08)	0.525
HC		0.343		0.380*
- 28 Weeks	− 0.12 (− 0.35, 0.11)	0.303	− 0.10 (− 0.34, 0.14)	0.425
- 36 Weeks	0.02 (− 0.19, 0.23)	0.878	0.03 (− 0.19, 0.25)	0.791
FL		0.392		0.369*
- 28 Weeks	−0.03 (− 0.09, 0.02)	0.244	− 0.02 (− 0.08, 0.04)	0.442
- 36 Weeks	− 0.00 (− 0.05, 0.05)	0.880	0.01 (− 0.04, 0.06)	0.729
EFW z score		0.900		0.901*
- 28 Weeks	−0.05 (− 0.18, 0.09)	0.476	− 0.06 (− 0.20, 0.09)	0.432
- 36 Weeks	−0.04 (− 0.17, 0.10)	0.567	− 0.05 (− 0.19, 0.09)	0.498
AC z score		0.865		0.832*
- 28 Weeks	−0.04 (− 0.18, 0.11)	0.603	− 0.05 (− 0.21, 0.10)	0.513
- 36 Weeks	−0.02 (− 0.17, 0.12)	0.749	−0.03 (− 0.18, 0.12)	0.668
BPD z score		0.909		0.810*
- 28 Weeks	0.11 (− 0.12, 0.35)	0.344	0.12 (−0.13, 0.36)	0.340
- 36 Weeks	0.10 (−0.07, 0.27)	0.239	0.09 (−0.09, 0.27)	0.336
HC z score		0.970		0.850*
- 28 Weeks	0.04 (−0.11, 0.19)	0.584	0.04 (−0.12, 0.21)	0.607
- 36 Weeks	0.04 (−0.11, 0.18)	0.600	0.03 (−0.13, 0.18)	0.736
FL z score		0.515		0.506*
- 28 Weeks	0.00 (−0.16, 0.17)	0.954	0.03 (−0.14, 0.20)	0.736
- 36 Weeks	0.07 (−0.09, 0.23)	0.407	0.10 (−0.07, 0.27)	0.268

Results are expressed as the expected difference (ratio) and 95% confidence intervals

Adjusted analyses including BMI category (< 30 vs ≥30), study centre, parity (0 vs ≥1), age at consent, smoking status and SEIFA IRSD quintile

* *p* value for test of time-by-log Leptin interaction to test whether the association between fetal ultrasound measure and log Leptin are different at 36 weeks to the association at 28 weeks

### Adiponectin

There were consistent associations identified between serum adiponectin concentrations and fetal ultrasound measures (Table 6).

**Table 6:** Relationship between log Adiponectin and Fetal ultrasound markers

Ultrasound Measure	Unadjusted Estimate (95% CI)	Unadjust *p* value	Adjusted Estimate (95% CI)	Adjust *p* value
EFW		0.010		0.008*
- 28 Weeks	−5.36 (−42.08, 31.35)	0.775	−8.77 (−45.68, 28.14)	0.641
- 36 Weeks	−94.09 (− 158.68, −29.51)	0.004	−100.85 (−164.98, −36.71)	0.002
SSFM		0.110		0.101*
- 28 Weeks	0.11 (−0.05, 0.28)	0.179	0.12 (−0.05, 0.30)	0.160
- 36 Weeks	−0.12 (− 0.38, 0.13)	0.343	− 0.12 (− 0.38, 0.13)	0.354
AFM		0.634		0.651*
- 28 Weeks	−0.05 (− 0.26, 0.16)	0.651	− 0.00 (− 0.21, 0.21)	0.988
- 36 Weeks	−0.13 (− 0.43, 0.17)	0.393	−0.08 (− 0.39, 0.23)	0.607
MTFM		0.688		0.517*
- 28 Weeks	0.00 (−0.23, 0.24)	0.970	−0.01 (− 0.25, 0.23)	0.943
- 36 Weeks	−0.11 (− 0.69, 0.46)	0.705	−0.20 (− 0.79, 0.39)	0.509
MTLM		0.035		0.013*
- 28 Weeks	0.09 (−0.11, 0.29)	0.377	0.09 (−0.12, 0.29)	0.405
- 36 Weeks	−0.33 (− 0.70, 0.03)	0.074	− 0.41 (− 0.77, − 0.05)	0.027
AC		0.012		0.010*
- 28 Weeks	−0.04 (− 0.31, 0.23)	0.768	− 0.04 (− 0.31, 0.23)	0.779
- 36 Weeks	−0.51 (− 0.82, − 0.21)	0.001	−0.53 (− 0.83, − 0.22)	<.001
BPD		0.056		0.056*
- 28 Weeks	0.06 (−0.00, 0.12)	0.055	0.04 (−0.02, 0.11)	0.176
- 36 Weeks	−0.02 (− 0.08, 0.04)	0.545	− 0.04 (− 0.10, 0.02)	0.244
HC		0.042		0.043*
- 28 Weeks	0.09 (−0.13, 0.30)	0.429	0.10 (−0.12, 0.32)	0.363
- 36 Weeks	−0.20 (− 0.41, 0.02)	0.071	− 0.18 (− 0.39, 0.03)	0.095
FL		0.061		0.088*
- 28 Weeks	0.03 (−0.02, 0.08)	0.272	0.02 (−0.03, 0.08)	0.406
- 36 Weeks	−0.03 (− 0.08, 0.02)	0.179	− 0.04 (− 0.08, 0.01)	0.160
EFW z score		0.986		0.938*
- 28 Weeks	−0.24 (− 0.37, − 0.10)	<.001	−0.23 (− 0.37, − 0.10)	<.001
- 36 Weeks	−0.24 (− 0.38, − 0.09)	0.001	−0.24 (− 0.38, − 0.10)	<.001
AC z score		0.427		0.371*
- 28 Weeks	−0.22 (− 0.36, − 0.08)	0.002	−0.21 (− 0.35, − 0.07)	0.004
- 36 Weeks	−0.30 (− 0.47, − 0.13)	<.001	−0.30 (− 0.46, − 0.13)	<.001
BPD z score		0.685		0.609*
- 28 Weeks	0.04 (−0.18, 0.26)	0.749	−0.01 (− 0.23, 0.21)	0.949
- 36 Weeks	−0.02 (− 0.19, 0.16)	0.861	−0.07 (− 0.26, 0.11)	0.434
HC z score		0.759		0.702*
- 28 Weeks	−0.10 (− 0.27, 0.06)	0.204	− 0.08 (− 0.24, 0.08)	0.316
- 36 Weeks	−0.13 (− 0.29, 0.02)	0.086	−0.12 (− 0.27, 0.03)	0.124
FL z score		0.530		0.645*
- 28 Weeks	−0.06 (− 0.23, 0.11)	0.514	− 0.07 (− 0.24, 0.11)	0.454
- 36 Weeks	−0.12 (− 0.29, 0.04)	0.150	−0.11 (− 0.28, 0.05)	0.176

Results are expressed as the expected difference (ratio) and 95% confidence intervals

Adjusted analyses including BMI category (< 30 vs ≥30), study centre, parity (0 vs ≥1), age at consent, smoking status and SEIFA IRSD quintile

* *p* value for test of time-by-log Adiponectin interaction to test whether the association between fetal ultrasound measure and log Adiponectin are different at 36 weeks to the association at 28 weeks

There were negative associations identified between serum adiponectin concentrations and measures of abdominal circumference (AC) and estimated fetal weight (EFW). Specifically, a 1-unit increase in *log* adiponectin concentration was associated with a reduction in mean AC of − 0.53 (− 0.83, − 0.22) millimetres (*p* < 0.001) and reduction in the mean EFW of − 100.85 (− 164.98, − 36.71) grams (*p* = 0.002) at 36 weeks’ gestation.

There were negative associations identified between serum adiponectin concentration and z scores for abdominal circumference (AC) and estimated fetal weight (EFW). Specifically, a 1-unit increase in *log* adiponectin concentration was associated with a reduction in the mean AC z score of − 0.21 (− 0.35, − 0.07) at 28 weeks (*p* = 0.004) and of − 0.30 (− 0.46, − 0.13) at 36 weeks (*p* < 0.001). Similarly, a 1-unit increase in *log* adiponectin concentration was associated with a reduction in the mean EFW z score of − 0.23 (− 0.37, − 0.10) at 28 weeks (*p* < 0.001) and of − 0.24 (− 0.38, − 0.10) at 36 weeks (*p* < 0.001).

There was a negative association identified between serum *log* adiponectin concentration and MTLM. Specifically, a 1-unit increase in *log* Adiponectin concentration was associated with a reduction in the mean MTLM of − 0.41 (0.77, − 0.05) millimetres at 36 weeks (*p* < 0.001).

### Time by Cardiometabolic interaction

The associations between serum *log* adiponectin concentration and mean EFW changed over time. At 28 weeks, there was a small and not statistically significant association and at 36 weeks, the association was larger in magnitude and statistically significant (*p* = 0.008).

The association between serum *log* Adiponectin concentration and mean AC changed over time. At 28 weeks, there was a small and not statistically significant association compared with at 36 weeks, the association was larger in magnitude and statistically significant (*p* = 0.01).

The association between serum *log* adiponectin concentration and mean HC changed over time, although neither individual associations were statistically significant. At 28 weeks, women with higher *log* adiponectin concentrations had fetuses with bigger head circumference, whereas at 36 weeks, women with higher *log* Adiponectin had fetuses with lower HC (*p* = 0.01).

The association between serum *log* adiponectin concentrations and mean MTLM changed over time. At 28 weeks, there was a small and not statistically significant association compared with at 36 weeks, the association was larger in magnitude and statistically significant (*p* = 0.013).

## Discussion

The main findings of this secondary exploratory analysis showed that increasing concentrations of adiponectin was associated with a reduction in abdominal circumference and estimated fetal weight, with this effect increasing over time. Furthermore, a higher triglyceride concentration was associated with an increase in abdominal circumference z score and estimated fetal weight at 36 weeks gestation. There were no apparent associations between inflammatory markers, fasting glucose, triglyceride and leptin concentrations with fetal ultrasound measurements.

To the best of our knowledge, this is the first study to describe the relationship between cardiometabolic biomarkers with fetal ultrasound measurements of biometry and adiposity. The current literature to date has reported on maternal or cord blood sampling and postnatal measurements of neonatal adiposity [41] or child growth trajectories [42]. There have been two large studies which have evaluated maternal cardiometabolic markers in the setting of a randomised control trials testing the effect of an antenatal dietary and lifestyle intervention [38, 43].

The strength of our analysis is the large sample size of 911 women and the reporting of fetal body composition as an outcome measurement. This study details an exploratory and hypothesis-generating analyses rather than confirmatory study. As a secondary study with a large quantity of statistical tests, any observed associations have a larger probability of being due to chance than indicated by the *p* value alone. Therefore, we did not account for the multiple comparisons. The limitation of this secondary analysis is the lack of a comparator group of women entering pregnancy with a normal BMI. Fasting measurements at 36 weeks for triglycerides and glucose were not obtained and this limited our interpretation to one time point only for these two cardiometabolic markers, although there is some literature to suggest that the impact of fasting versus non-fasting samples may not be as great as initially thought.

The main finding of our secondary analysis relating to adiponectin is consistent with the current literature. In women entering pregnancy with a normal body mass index, Lekva and associates found a reduction in adiponectin concentrations in the 3rd trimester, and this occurred independently of body mass index and maternal insulin resistance [22]. Low adiponectin concentration is associated with a higher prevalence of newborns classified as large for gestational age and increased birthweight [22]. Regarding interventions during pregnancy, the LIMIT trial showed that a dietary and lifestyle intervention did not change the concentrations of the cardiometabolic biomarkers in women who were overweight and obese [38]. The Fit for Delivery intervention in low risk women [44] showed a reduction in insulin and leptin concentrations, but this did not reduce the incidence of gestational diabetes, the primary outcome.

While adiponectin concentrations do not alter with dietary change, there is increasing interest in the supplementation of adiponectin has promising applications in the adult populations [18, 22, 24]. In vivo and in vitro studies [2] have shown that adiponectin supplementation in pregnancy may alter fetal growth through improving insulin sensitivity [45]. The proposed mechanism relates to the down regulation of key placental nutrient transporters within the syncytiotrophoblasts, including amino acid transporters such as System A [22, 45]. Adiponectin as a therapy may reduce cardiovascular risk in the non-pregnant overweight and obese mouse model [18]. Further studies with experimental animal models along with clinical applications are required.

Interestingly, leptin did not show any consistent effect on fetal growth or adiposity in our study. This was supported by a recent study by Castro who performed maternal serum leptin sampling after delivery (to reduce the effect of placental leptin production), and found no association with neonatal adiposity [46]. Josefson measured the concentrations at 36 weeks gestation and found an association with neonatal adiposity [47]. This highlights that each cardiometabolic marker has a different pattern during pregnancy and the timing of sampling may impact on the interpretation of results. Interestingly, fetal exposure to leptin along with high cord blood concentrations, have been positively associated with birthweight, neonatal adiposity, postnatal and childhood growth trajectories [42].

Maternal triglyceride concentration at 36 weeks was associated with an increase in z scores for abdominal circumference and estimated fetal weight. This is consistent with studies from women with gestational diabetes, where fetal growth and adiposity correlate with maternal triglyceride levels, independent of body mass index [9]. The exact role of maternal triglycerides [48], lipoprotein receptors, binding proteins and lipases and the placental flow of maternal fatty acids [9] in the setting of obesity remains unclear [48] and further studies are required.

The relationship between maternal inflammatory markers and fetal adiposity is also interesting. There is evidence to support that maternal obesity increases pro-inflammatory cytokines [49], which in turn has been shown to stimulate the inflammatory pathways within the placenta [50, 51]. The effect of the inflammatory milieu related to maternal obesity on the fetus [52], newborn [53] and child [54] remains unclear [55, 56] and requires further evaluation. A reason for the lack of effect seen in this study may relate immune modulation in pregnancy, which may dampen down the chronic, low grade inflammatory response related to obesity [57].

In this study, maternal concentration of glucose at 28 weeks was not associated with fetal body composition. While the findings of the HAPO study found that a modest increase in maternal glucose levels was associated with an increase in birth weight [58], the HAPO study included women with a normal BMI. The effect of obesity has a more significant, stronger and long term effect on the risk of large for gestational age infants [59] when compared to pregnancy mediated insulin resistance, present from 28 weeks onwards [58].

Understanding of the mechanisms and timing of critical fetal growth changes represents an evolving area of obesity related research. From a public health perspective, the only preventive strategy to reduce the intergenerational transmission of obesity [60] is to optimise maternal weight and reduce obesity related morbidity prior to pregnancy. Current studies are underway to assess dietary and lifestyle interventions to reduce maternal obesity prior to pregnancy or in early pregnancy [61]. Further research is required to assess the role of adiponectin and supplementation in the setting of obesity in pregnancy [17].

## Conclusion

Our exploratory study has contributed to the further understanding of the fetal overgrowth pathway. High concentrations of adiponectin were found to be associated with a reduction in abdominal circumference and estimated fetal weight in women who are overweight or obese. Adiponectin is a promising biomarker that may have a role in the modulation of fetal growth in the future.

## Data Availability

The data that support the findings of this study are available from the LIMIT Randomised Trial group but restrictions apply to the availability of these data, which were used under license for the current study, and so are not publicly available. Data are however available from the authors upon reasonable request and with permission of LIMIT Randomised Trial group.

## Abbreviations

AA: Abdominal area
AC: Abdominal circumference
AFM: Abdominal fat mass
BMI: Body Mass Index
BPD: Biparietal diameter
EFW: Estimated Fetal Weight
FL: Femur length
HC: Head circumference
MTFM: Mid-thigh fat mass
MTLM: Mid-thigh lean mass
SSFM: Subscapular fat mass
